# Study of Dynamic Failure Behavior of a Type of PC/ABS Composite

**DOI:** 10.3390/ma17081728

**Published:** 2024-04-10

**Authors:** Jiayu Zhou, Zhaodong Xia, Dongfang Ma, Huanran Wang

**Affiliations:** 1 Key Laboratory of Impact and Safety Engineering, Ministry of Education, Ningbo University, Ningbo 315211, China; zsetelle5@gmail.com (J.Z.); xiazhaodong2024@163.com (Z.X.); wanghuanran@nbu.edu.cn (H.W.); 2 College of Science & Technology, Ningbo University, Ningbo 315300, China

**Keywords:** failure analysis, polymers and plastics, Johnson–Cook constitutive, numerical simulation, inversion method

## Abstract

PC/ABS composites are commonly used in airbag covers. In this paper, uniaxial tensile experiments of a PC/ABS composite at different temperatures and strain rates were conducted. The results showed that the temperature and loading rate affect the mechanical properties of the PC/ABS composite. As the temperature increases, the yield stress decreases and the strain at the moment of fracture increases, but the strain rate at the same temperature has a relatively small effect on the mechanical properties, which are similar to ductile materials. The experimental results were applied to the Abaqus model which considered thermal effects and the exact Johnson–Cook constitutive parameters were calculated by applying the inverse method. Based on the constitutive model and the failure analysis findings acquired by DIC, the uniaxial tensile test at the room temperature and varied strain rates were simulated and compared to the test results, which accurately reproduced the test process. The experiment on target plate intrusion of the PC/ABS composite was designed, and a finite-element model was established to simulate the experimental process. The results were compared with the experiments, which showed that the constitutive and the failure fracture strains were valid.

## 1. Introduction

In recent decades, polymers have become increasingly popular in various industries due to their superior properties, such as high specific strength and low cost. ABS (acrylonitrile-butadiene-styrene) has a good durability and rigidity, containing rubber particles which allow it to suffer more plastic deformation under impact loads [1]. PC (polycarbonate) is often used in structural support due to its wide temperature and impact resistance [2]. TC-45M is a composite material made of a mixture of PC and ABS, which exhibits better impact toughness and tensile strength compared to pure polymer [3,4,5]. It has gradually replaced PC materials and is extensively used in the automotive industry for airbag covers. The study of its failure behavior can provide effective theoretical support for the study of vehicle safety performance.

During the last few decades, uniaxial tensile or compressive testing has represented a widely used method for studying polymer mechanical properties at different temperatures and strain rates Zheng et al. performed uniaxial stretching of Poly-Ether-Ether-Ketone at elevated temperatures and simulated its deformation behavior using a phenomenological model named DSGZ [6]. A study on the uniaxial compressive deformation behavior of PC/ABS blends at different rates and temperatures was conducted by Wang et al. [7]. They utilized a modified DSGZ model to characterize the deformation after unloading and reloading. Louche et al. conducted uniaxial tensile experiments on ABS polymer materials at various strain rates and temperatures to investigate their performance under impact loading, simulated the experimental process using the J-C constitutive (Johnson–Cook constitutive) to simulate the experimental process, and finally compared the experimental and numerical results to prove the correctness of the model [8]. Based on uniaxial tensile tests and single-edge-notch-tension (SENT) tests, two rubber-toughened thermoplastic polymer blends with different volume fractions of PC and ABS were analyzed experimentally, as well as by constitutive models and finite-element simulations with regard to their large strain deformation and fracture behavior by Hund et al. [9]. The impact behavior and modeling of ABS and polybutylene-terephthalates (PBT) were obtained as a function of impact velocity and temperature from a multiaxial impact test by Duan et al., and the deformation and failure of polymers were analyzed using a combination of experiments and finite-element analysis [10,11].

When a car is subjected to a violent impact force, airbag ejection leads to an impact loading, and the cover plant temperature increases [12]. However, there are limited thermodynamic constitutive models available for impact loading and the viscoplasticity model which considers temperature, suffers from the issue of too many parameters. In this paper, a uniaxial tensile test of the PC/ABS composite was conducted. Based on the experimental results of middle and low strain rates, the initial parameters of the J-C constitutive were obtained by MATLAB fitting. It should be noted that the parameters determined in this way may have some inaccuracy [13,14,15]. Subsequently, the initial parameters were substituted into Abaqus 2020 to simulate the experimental process, and the parameters were gradually adjusted to invert the modified constitutive model, and the results have a high accuracy [16,17,18], and apply to the high strain rate case. Based on the determined constitutive model and fracture parameters, numerical simulations of high-speed tensile testing of PC/ABS were conducted and compared with experimental results. Finally, a target plate impact experiment for the PC/ABS composite was designed, and a finite-element model was established to simulate the experimental process. The results were compared with the experiments, indicating the validity of the constitutive model and the failure fracture strain.

## 2. Materials and Methods

### 2.1. Material

The material used in the experiments of this paper is the PC/ABS composite. The PC/ABS composite material can combine the excellent properties of PC and ABS, improve the heat resistance, impact resistance and tensile strength of ABS, reduce the cost of PC and the viscosity of the melt, improve the processing performance, and reduce the internal stress of the product and the sensitivity of the impact strength to the thickness of the product. In addition, it also has a low price, low density, and other characteristics. The PC/ABS can be used as a structural material. It has been widely used in the car tool industry as a car cover.

A composite is not a simple blend of many materials. Therefore, it is not possible to derive accurate results of PC/ABS failure behavior from a single ABS or PC [19]. To accurately simulate the failure behavior of automotive covers during vehicle impacts, the material behavior at different temperatures and strain rates is investigated in this paper.

### 2.2. Uniaxial Tensile Test of the PC/ABS Composite Material

**Figure 1:**
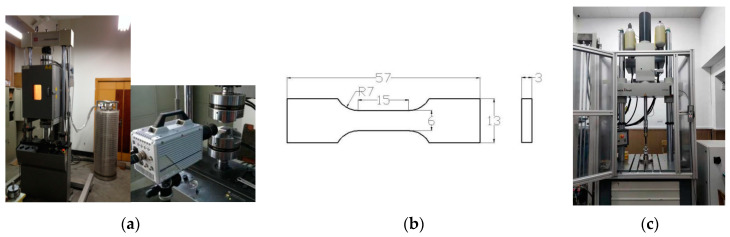
Design of uniaxial tensile experiment: (**a**) MTS-810 dynamic and static materials testing machine; (**b**) Size of test material (unit: mm); (**c**) ZwickRoell-5020 high-speed hydraulic tensile testing machine system.

The PC/ABS composite model TC-45M (Dongguan Xinrui polymer material Technology Co., Ltd., Dongguan, China) was used in this study, and a specimen of the PC/ABS composite was designed for testing in the MTS-810 dynamic and static materials testing machine (MTS Systems Corporation, Eden Prairie, MN, USA), as shown in Figure 1a. The MTS-810 has a measured strain rate range of 10^−4^–10 s^−1^. The low and medium strain rate tensile test at various temperatures can be realized with the temperature chamber. The influence of specimen size on the force balance error should be considered in the case of high-speed drawing [20]. The specific dimensions of the specimen are shown in Figure 1b. The specimen length of 15 mm can greatly reduce the influence of specimen size on the results of high-speed tensile experiments and can be used in quasi-static experiments too [21].

The MTS-810, shown in Figure 1a, was used to perform uniaxial tensile tests on the PC/ABS composite at various ambient temperatures and strain rates. The ambient temperature was accurately controlled by a temperature chamber (238.15 K was achieved by continuously passing liquid nitrogen into the chamber). The PC/ABS composite is mostly used in automobile manufacturing, so its failure behavior under a high strain rate is also the focus of this paper. The ZwickRoell-5020 high-speed hydraulic tensile testing machine system (ZwickRoell GmbH & Co. KG, Ulm, Germany) was selected, shown in Figure 1c, which can also cooperate with the temperature chamber. This device has a strain rate measurement range of 10–1000 s^−1^. The sample still uses the specifications shown in Figure 1b. The above experimental conditions are listed in Table 1.

### 2.3. PC/ABS Composite Material Ballistic Impact Test

**Figure 2:**
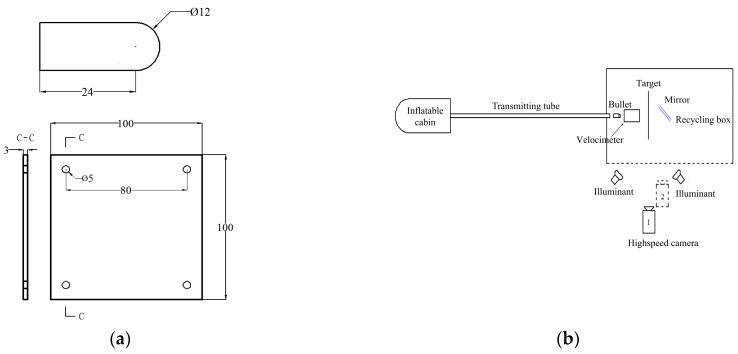
Ballistic impact test experimental design diagrams: (**a**) Design dimensions of target plate and bullet (unit: mm); (**b**) Layout of penetration test device.

The PC/ABS composite material penetration test sample was designed as a 100 mm×100 mm×3 mm rectangular target plate. Four holes were punched into the sample’s four corners to make it unmovable. The bullet used in the experiment is a cylindrical length of 24 mm with a hemispherical head measuring 12 mm in diameter. The bullet is made of Cr12MoV tool steel. Figure 2a provides the plate’s and bullet’s precise design.

The experiment of the ballistic impact test uses a high-speed air gun in combination with a high-speed camera system. Figure 2b displays the schematic of the experimental device arrangement. The caliber of the high-speed air gun launch tube is 12 mm, and the length of the gun tube is 4 m. A tachymeter was placed between the high-speed air gun barrel and the test simple to measure the average velocity of the bullet strikes. After research, it was found that the average speed of cars on the highway was approximately 30 m/s. To test the applicability of the PC/ABS composite material, the speed of the experiment should be greater than 30 m/s [22]. After many empty gun experiments, with the same bullet and the conditions of the pressure, the bullet’s hitting speed is maintained at around 34 m/s.

The bullet penetration process is captured by a high-speed camera system. The camera is pointed toward the side of the target plate, and the camera’s shooting direction is perpendicular to the path of the bullet’s incidence. A mirror was placed on the target’s rear to capture back-view pictures during the bullet impact, positioned at a 45° angle to both the direction of the bullet and the direction of high-speed photography. This allowed the high-speed camera to capture images of the deformation and fracture on the specimen’s back. In all experiments, the sampling frequency of the high-speed camera was set at 4 × 10^4^ Hz, the time interval between neighboring photos was 25 μs, and the shooting resolution was set at 384 × 288 pixels.

## 3. Results and Discussion

### 3.1. Uniaxial Tensile Test Results

**Figure 3:**
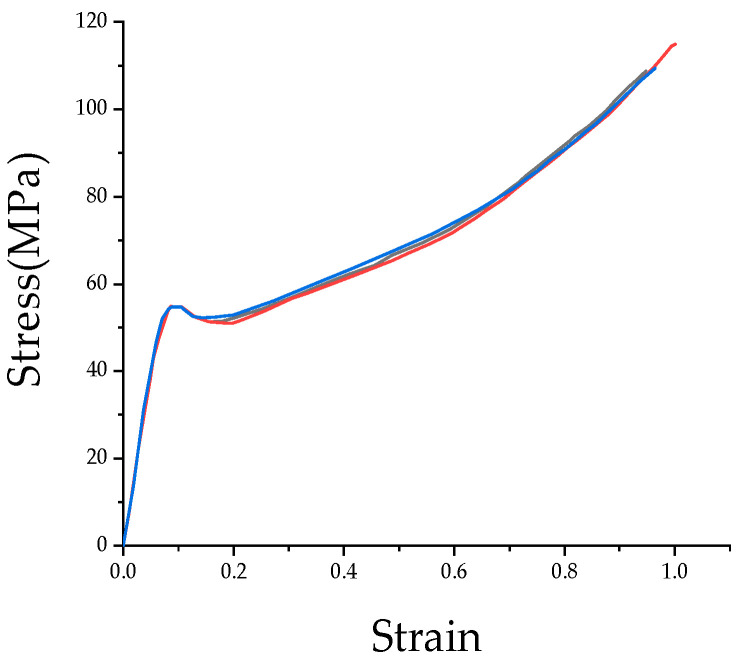
Three times quasi static test results of the PC/ABS composite under 273.15 K and a strain rate of 0.01 s^−1^.

**Figure 4:**
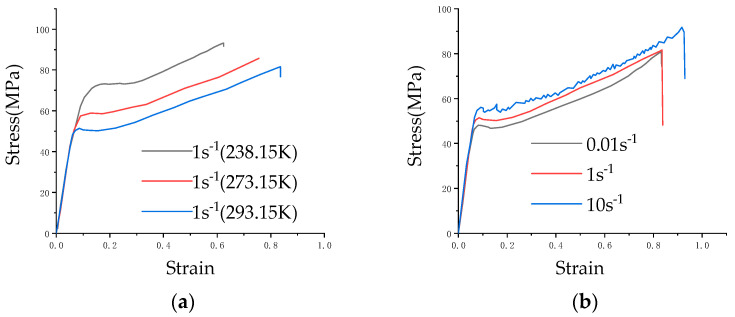
Effect of a single variable for the failure behavior of the PC/ABS composite: (**a**) Force–displacement curve of the PC/ABS composite under the same strain rate (1 s^−1^) and different temperatures; (**b**) Force–displacement curve of the PC/ABS composite under different strain rates at room temperature.

Uniaxial tensile tests were conducted on the MTS-810 testing machine at varying temperatures and strain rates. Uneven material distribution can lead to unrepeatable experimental results, so this paper performs three replications under each experimental condition to assess the homogeneity of the composite [23]. The typical results are displayed in Figure 3, which illustrates the composite’s good homogeneity. Figure 4a,b depict the results of averaging three sets of test results for different ambient temperatures at the same strain rate, as well as different strain rates at the same ambient temperature.

The figure shows that the mechanical properties of the PC/ABS composite are influenced by both the loading rate and ambient temperature. The temperature factor responds to these mechanical properties in a particularly noticeable way: as the ambient temperature rises, yield stress and fracture strain respectively decrease and increase. The mechanical properties’ effects are relatively weak by the strain rate at the same temperature and the mechanical properties are similar to those of traditional ductile materials [24,25].

**Figure 5:**
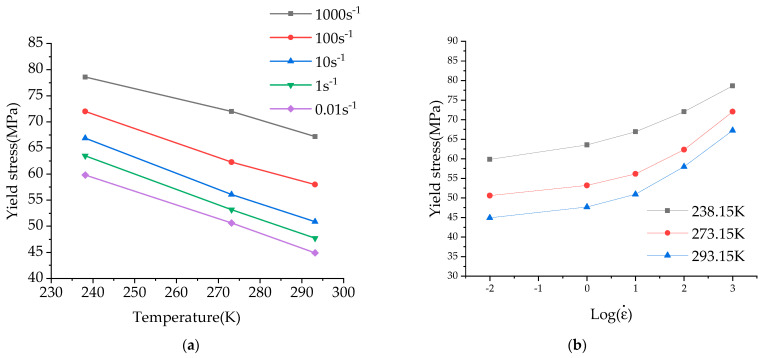
Comparison of yield stress under different experimental conditions: (**a**) Yield stress temperature curve; (**b**) Yield stress strain rate (logarithmic) curve.

The yield stresses at different temperatures and strain rates are compared and analyzed as shown in Figure 5. The results show that the yield stress of the material is linear with the temperature and the logarithm of strain rate. The yield stress of the material increases with the increase in strain rate and decreases with the increase in temperature. When the temperature rises, the movement of polymer chains in the PC/ABS composite is activated, and under the action of external load, the molecular chains are rotated and displaced, the plastic flow is strengthened, and the yield stress is reduced. Under quasi-static tensile conditions, the material is in a steady state, allowing infinite plastic flow; under medium to high strain rates, the polymer chains in the PC/ABS composites cannot rotate and displace rapidly, resulting in the strengthening of the yield stress.

### 3.2. Experimental Results of Ballistic Impact Test

**Figure 6:**
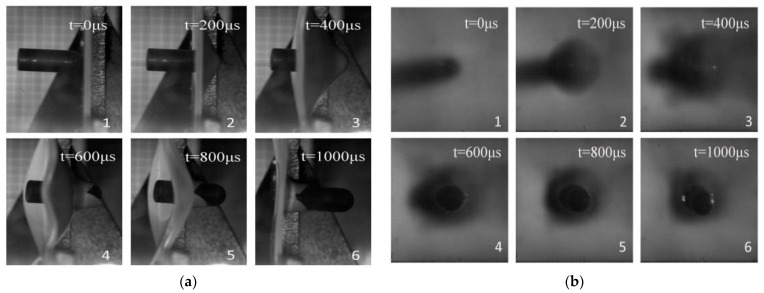
Penetration process under different angles of view of the experiment: (**a**) Penetration process under the side angle of view of the experiment; (**b**) Penetration process from the experimental back view.

Target plate penetration experiments were conducted on the PC/ABS composite under the required experimental conditions. The high-speed impact process was recorded by a high-speed camera. For the convenience of observation, the instant of bullet impact (when the bullet was just in touch with the target plate) was defined as the start time. Figure 6 displays the experimental processes of the penetration procedure from the side and rear perspectives. It can be found that it has a large deformation and a long plastic stage during the experiment, rather than a brittle fracture. Therefore, it is found that the PC/ABS composite material is a typical ductile material by analyzing the deformation and failure mode of the target plate.

### 3.3. Determination of the Parameters of the J-C Constitutive

The number of viscoelastic thermodynamic coupling models under impact loads is small, and there are many problems such as too much parameter measurement [26,27,28]. Because PC/ABS is widely used in the preparation of vehicles, the study of its failure behavior should pay attention to the influence of strain rate and temperature, especially the plastic deformation and failure at a high strain rate, rather than its creep or relaxation behavior. Therefore, the J-C constitutive is selected in this paper. The J-C constitutive model is a phenomenological model that describes plastic hardening, strain rate effects, and thermal softening of materials [29]. These three phenomenological formulations are connected multiplicatively in the J-C constitutive. The J-C constitutive is mainly applied to materials with large deformations, high strain rates, and high temperatures, meaning it is suitable for numerical simulations of most materials. The form of Equation (1) is as follows [30,31,32]:

(1) $$\sigma =(A+B\varepsilon_{p}^{n})(1+C\ln\frac{\dot{\varepsilon }}{{\dot{\varepsilon }}_{0}})[1-{\left(\frac{T-T_{0}}{T_{m}-T_{0}}\right)}^{m}]$$

where $\varepsilon_{p}$—equivalent plastic strain; $\dot{\varepsilon }$—equivalent plastic strain rate; ${\dot{\varepsilon }}_{0}$—reference strain rate; $T_{0}$—reference temperature; $T_{m}$—melting point temperature of the material; T—test temperature. A, B, C, n, m are the material parameters.

**Figure 7:**
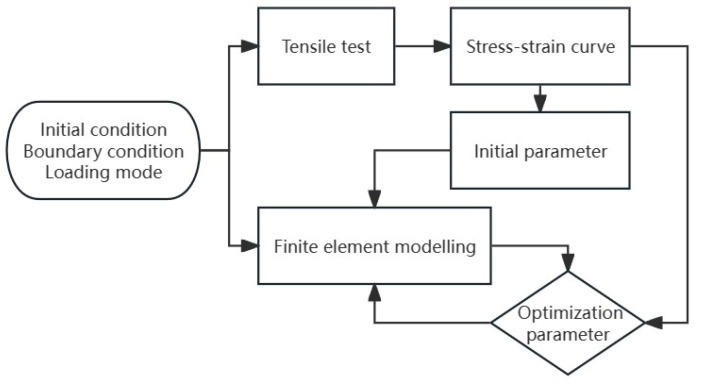
Process of determining parameters by the inversion method.

The following is the principle used in this research to determine the parameters A,B,C,n,m of the J-C constitutive: The stress–strain curve transformed by the force–displacement curve at the reference temperature of 238.15 K and strain rate of 0.01 s^−1^ gives the values of A, B and n, the stress–strain curve transformed by the various strain rates at 238.15 K gives the value of C; the stress–strain curve transformed by the various temperatures at 0.01 s^−1^ gives the value of m. Based on this principle, the initial parameters of the J-C constitutive are determined as A=60 MPa, B=115 MPa, C=0.03, n=1.75, m=0.95. However, the parameters determined by this method have a large error and cannot accurately reproduce the failure behavior of the material [15]. The constitutive model is mainly used in the field of numerical simulation, so researchers will reproduce the experimental process by numerical simulation and modify the parameters by comparing with the results of uniaxial tensile experiment. The above parameters are put into the Abaqus 2020 to apply the same loading conditions as the test. Then, numerical simulation is compared with the tests, and the parameters are adjusted until they are in total agreement with the test. The specific process of determining the final parameters is shown in Figure 7. This method is hereafter referred to as the inversion method. The basic mechanical parameters of the PC/ABS composite are as follows: the density is 1120 kg/m^3^, the modulus of elasticity is 1750 MPa, Poisson’s ratio is 0.38, and the specific heat capacity is 1400 J/(kg·K). The Abaqus solver was used for numerical simulation, and the mesh type was C3D8R. After many attempts, the mesh size had no obvious influence on the simulation results.

**Figure 8:**
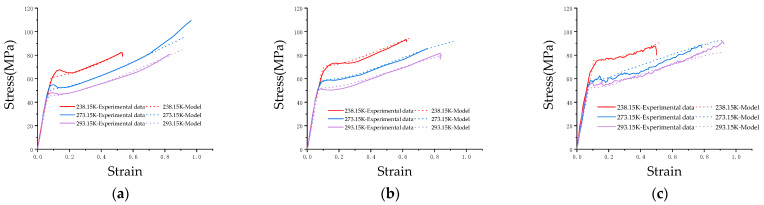
Comparison between numerical simulation and experiment after modification by inversion method: (**a**) Test results with strain rate of 0.01 s^−1^; (**b**) Test results with strain rate of 1 s^−1^; (**c**) Test results with strain rate of 10 s^−1^.

Through the above inversion method, J-C constitutive parameters are determined and shown in Table 2. A comparison between numerical and experimental results is shown in Figure 8, and it should be pointed out that there is no failure criterion, so there is no steep drop in the numerical simulation curve. At this time, the deformation process can be basically reproduced at low and medium strain rates.

### 3.4. Validation of J-C Constitutive Parameters

**Figure 9:**
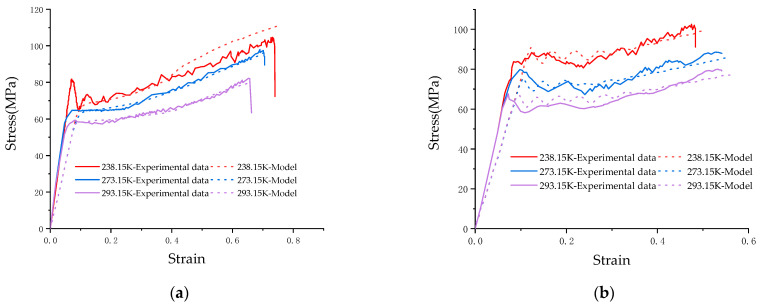
Experiments and simulations at high strain rates validate the accuracy of the constitutive model: (**a**) Comparison of results under different temperatures with strain rate of 100 s^−1^; (**b**) Comparison of results under different temperatures with strain rate of 1000 s^−1^.

The results obtained using the inversion method are only applicable to low and medium strain rates. However, the cover material is often subjected to high-speed impact loading when the airbag is deployed. To test the validity of the J-C constitutive determined by the inversion method when applied to high strains, we calibrated it with high strain rate tensile tests. Using a ZwickRoell-5020 high-speed tensile tester, we performed uniaxial tensile tests at strain rates of 100 s^−1^ and 1000 s^−1^ at three ambient temperatures sustained by particular temperature chambers. The test results were compared with the J-C constitutive numerical simulation data obtained using the inversion approach. The numerical simulation results of the J-C constitutive were close to the test results. Figure 9 presents the comparative results. This shows that the J-C constitutive derived from the inversion approach is fit for large strain rates. It should especially be pointed out that the experimental process can be regarded as an adiabatic process under high-speed impact [33], and a large amount of heat will be generated in the experiment, and a large degree of temperature change will be generated in the pattern. Therefore, a mechanical thermal effect is added in the simulation to correct for the effects of adiabatic warming.

### 3.5. Failure Behavior Analysis in One-Dimensional Tensile State

**Figure 10:**
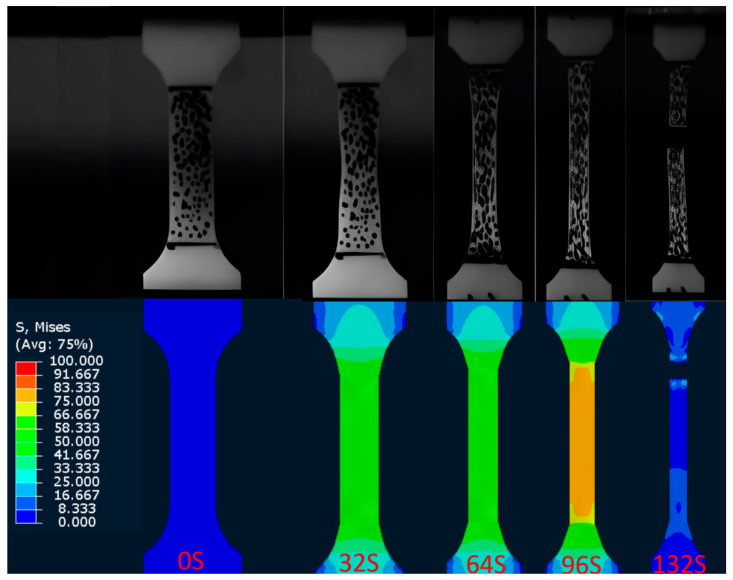
Comparison between the deformation process of numerical simulation specimen at different times and the record of the high-speed camera.

The J-C constitutive parameters constructed above do not include a failure criterion. Researchers are concerned with the failure behavior of materials in engineering. The failure behavior of this composite material is examined in this chapter. A high-speed video camera recorded the deformation and fracture process of the PC/ABS composite at room temperature with different strain rates. Note that other temperatures were achieved by an ambient temperature box. Therefore, the process for other temperatures could not be captured with a high-speed video camera. Figure 10 shows typical results. Digital image correlation (DIC) can be used to measure dimensional changes in drawing patterns in real-time using optical sensors. The deformation information of the specimen under tension at different strain rates and the local deformation of the specimen at the failure time was obtained with a DIC, and the fracture strain $\varepsilon_{tr}$ is calculated (Equation (2) shows the calculation, where $A_{0}$ represents the original cross-sectional area of the material, and A represents the cross-sectional area of the material fracture) [34]. The local strains in the failure region are listed in Table 3.

(2) $$\varepsilon_{tr}=\ln\left(\frac{A_{0}}{A}\right)$$

**Figure 11:**
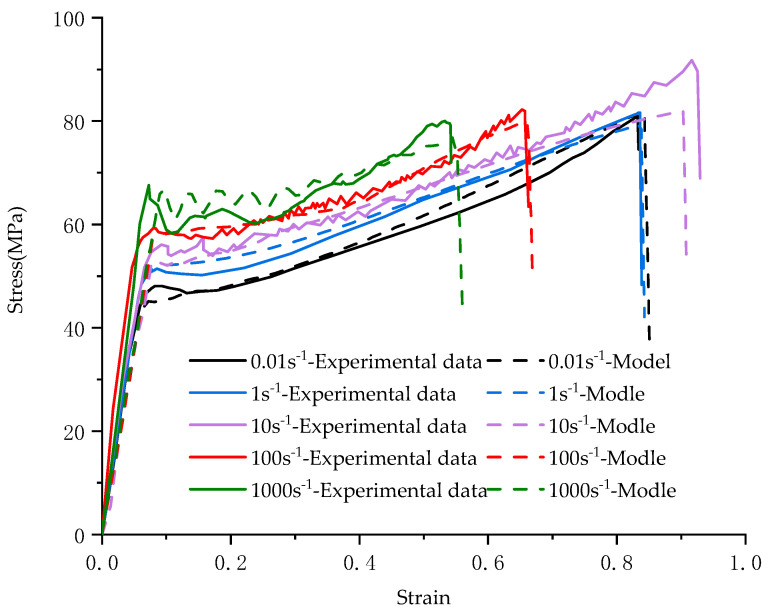
Comparison between uniaxial tensile test results and numerical simulation of the PC/ABS composite at room temperature and different strain rates.

In this paper, based on the J-C constitutive constructed by the inversion method combined with the fracture strain $\varepsilon_{tr}$ calculated by the deformation information of the specimen. Numerical simulation was conducted to analyze the uniaxial tensile behavior of the PC/ABS composite at room temperature (293.15 K) under different strain rates. The simulation compares the process to the recording of a high-speed camera. Figure 10 displays the typical comparison results, while Figure 11 compares the force–displacement curves of the tests under different strain rates with the numerical simulations. It can be seen from the figures that the J-C constitutive determined by the inverse method and the failure parameters obtained by using DIC can reproduce the uniaxial tensile test process of the PC/ABS composite under different strain rates at room temperature.

The J-C failure model is shown in Equation (3) [35], which describes the effects of stress triaxiality, strain rate, and temperature in a decoupled form so that factors can be removed when they are not important to the study.

(3) $$\varepsilon_{tr}=[D_{1}+D_{2}\exp D_{3}(\frac{\sigma_{m}}{\sigma_{eq}})][1+D_{4}\ln\frac{\dot{\varepsilon }}{{\dot{\varepsilon }}_{0}}][1+D_{5}(\frac{T_{s}-T_{r}}{T_{m}-T_{r}})]$$

where $\sigma_{m}$—hydrostatic stress; $\sigma_{eq}$—equivalent strength; $T_{s}$—sample temperature; $T_{r}$—reference temperature. It should be noted that the temperature of the sample during the stretching process will change significantly due to the generation of a large amount of heat, so $T_{s}$ refers to the internal temperature of the material before the sample fracture, rather than the ambient temperature.

Polymer materials are highly sensitive to temperature [36], and during the tensile process, a large amount of heat is generated in the sample, leading to a temperature rapid increase in material. To accurately analyze failure behavior, the failure temperature in the finite-element simulation is chosen as the specimen’s fracture temperature. This paper focuses on the effect of strain rate and temperature on the failure behavior of the PC/ABS materials, for which the J-C failure model is degraded as shown in Equation (4).

(4) $$\varepsilon_{tr}=[d_{1}+d_{2}\ln\frac{\dot{\varepsilon }}{{\dot{\varepsilon }}_{0}}][1+d_{3}(\frac{T_{s}-T_{r}}{T_{m}-T_{r}})]$$

where ${\dot{\varepsilon }}_{0}=0.01\;s^{-1}$, $T_{r}=293\;K$, $T_{m}=450\;K$. After fitting, it can be determined that $d_{1}=0.825,\;d_{2}=-0.043,\;d_{3}=2.6$. At medium strain rates, thermal softening takes a dominant role and the temperature rises rapidly, leading to a slight increase in the fracture strain. However, at high strain rates, high-velocity impacts dominate the fracture strain of the material, the fracture strain decreases, heat fails to accumulate in the material in large quantities, and the temperature rise before fracture is relatively few.

$T_{S}$ is shown in Table 3, and its theoretical temperature can also be calculated from Equation (5) [37], where density $\rho =1120{\;\mathrm{kg}/m}^{3}$, specific heat capacity c=1400 J/(kg·K) and mechanical thermal effect β=0.9, and ΔT is the elevated temperature. At a strain rate of 0.01 s^−1^, the sample is in thermal equilibrium with the outside world and the $T_{S}$ can be regarded as the same as the ambient temperature.


(5) $$\Delta T=\frac{\beta \int_{0}^{\varepsilon_{tr}}\sigma d\varepsilon_{tr}}{c\rho }$$

### 3.6. Failure Behavior Analysis under Ballistic Impacts

The ballistic impact experiment was numerically simulated. The inputs were the DIC-calculated failure fracture strain and the J-C constitutive parameters. A friction coefficient of 0.25 was used to describe the interaction between the bullet and the target plate.

**Figure 12:**
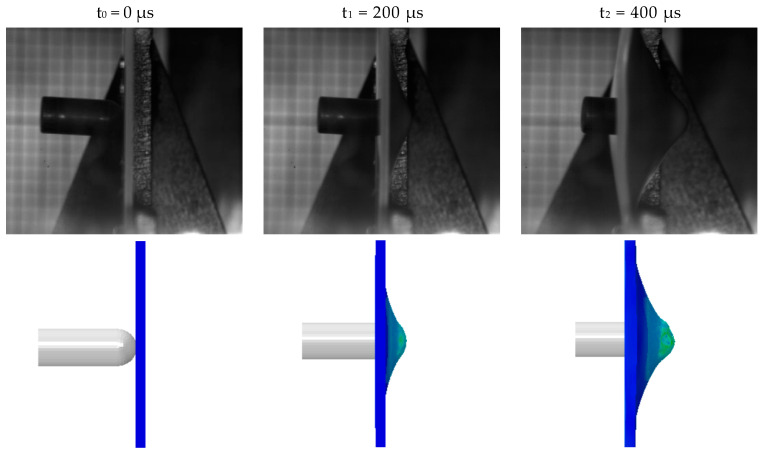

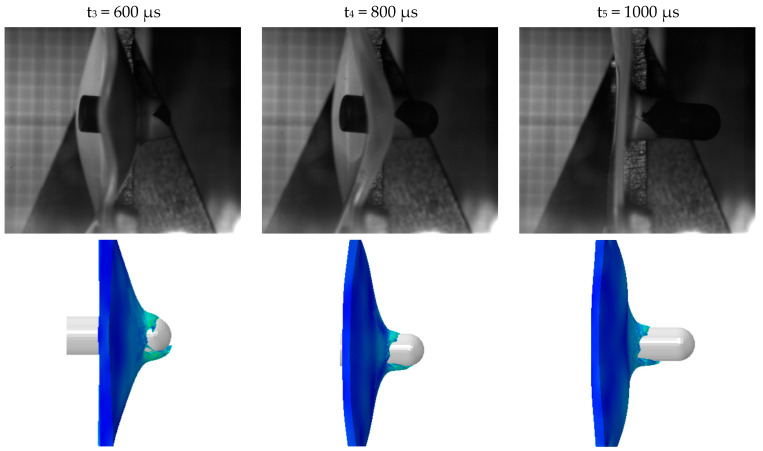
Comparison of the penetration failure process of the target plate in simulation and experiment.

**Figure 13:**
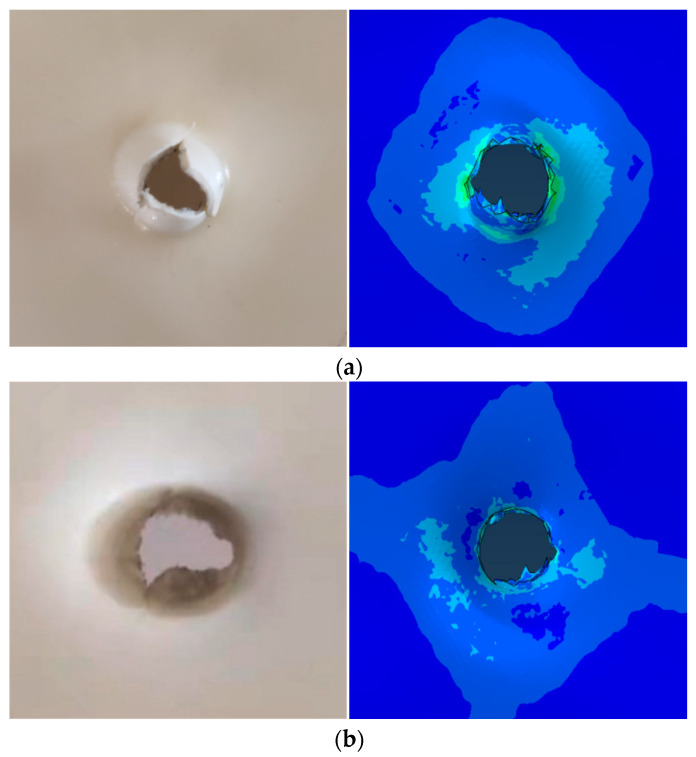
Comparison between the failure shape of the target recovered in the penetration experiment and the simulation results: (**a**) Comparison between the experiment on the back of the target and the simulation results; (**b**) Comparison of simulation results of the frontal experiment of the target plate.

Figure 12 shows the simulation results of the finite-element simulation of the PC/ABS composite for the penetration experiment, as well as a comparison to the experimental process of penetrating the target plate. Time (t_0_ = 0 μs) was defined as the moment the bullet began contact with the target plate. Then, we compared the simulation with the target plate’s damage shape and the bullet’s location at each of the following times: t_0_ = 0 μs, t_1_ = 200 μs, t_2_ = 400 μs, t_3_ = 600 μs, t_4_ = 800 μs, and t_5_ = 1000 μs. The results of the finite-element simulation were found to be in good agreement with the experimental results. The finite-element simulation’s damage shape and experimentally reclaimed target plate were compared, as shown in Figure 13. The finite-element simulation results can be accepted given the intricacy of the experimental procedure, the target plate fixation error, and several irresistible factors like air pressure instability, bullet ejection deviation, etc., [38]. In conclusion, the failure fracture strain computed by the deformation information of the specimen and the J-C constitutive parameters of the PC/ABS composite established using the inversion method are both accurate in this paper.

## 4. Conclusions

PC/ABS composites were subjected to uniaxial tensile tests at different speeds and temperatures. A high-speed video camera filmed the samples’ deformation process while they were at room temperature. Based on the experimental results, this paper used the inverse method to determine the J-C constitutive model that can be used to describe such a composite. The high-speed impact tensile tests of the PC/ABS composite were simulated numerically using Abaqus 2020 and the determined constitutive model and compared with the test results. In addition, the numerical simulation can reproduce the test process recorded by a high-speed camera in combination with the failure parameters obtained from the DIC analysis. We carried out the design of target penetration experiments simulation. The penetration simulation reproduces the experimental process and the failure model is correct. The main conclusions are as follows:

(1) The results of tensile tests at different temperatures and different strain rates show that such composite have more obvious temperature effect and strain rate effect, the yield stress decreases with the increase in temperature and increases with the increase in strain rate. The yield stress is linearly dependent on temperature and the logarithm of the strain rate;
(2) The J-C constitution established by the inversion method in this paper has high accuracy and is applicable to the PC/ABS composite. The failure behavior of the material at different temperatures and strain rates can be predicted;
(3) Based on the local deformation of the sample recorded by the DIC technique, the fracture strain of the PC/ABS composite can be deduced. This fracture strain can accurately reproduce the fracture behavior of uniaxial tensile materials.

## Figures and Tables

**Table 1:** Uniaxial tensile test conditions.

Strains (s^−1^)	Temperature (K)	Equipment Model	Loading Rate (mm/s)
0.01	238.15/273.15/293.15	MTS-810	0.15
1			15
10			150
100		ZwickRoell-5020	1500
1000			15,000

**Table 2:** J-C constitutive parameters of the PC/ABS composite.

A	B	C	n	m
57.5 MPa	120 MPa	0.032	1.734	1.02

**Table 3:** Fracture strains and sample temperature.

**strain rate (s^−1^)**	0.01	1	10	100	1000
**fracture strain**	0.825	0.833	0.922	0.646	0.527
**sample temperature by simulation (K)**	293	313	328	311	309
**sample temperature by theory (K)**	293	320.6	326.5	313.9	312.1

## Data Availability

The raw/processed data required to reproduce these findings cannot be shared at this time as the data also form part of an ongoing study.
